# Oxytocin infusion dose-response to maintain uterine tone in obese elective cesarean patients: a randomized controlled trial

**DOI:** 10.3389/fphar.2024.1361953

**Published:** 2024-04-17

**Authors:** Qin-Fang Yan, Ling Ai, Yi-Min Huang, Jianguo Wang, Fei Xiao, Huiqin Xu, Xue-Dong Tang

**Affiliations:** ^1^ Department of Obstetrics and Gynecology, Jiaxing Women and Children's Hospital of Wenzhou Medical University, Jiaxing, China; ^2^ Department of Center Lab, Jiaxing Women and Children's Hospital of Wenzhou Medical University, Jiaxing, China; ^3^ Department of Anesthesia, Jiaxing Women and Children's Hospital of Wenzhou Medical University, Jiaxing, China

**Keywords:** cesarean section, obesity, oxytocin, PostPartum Hemorrhage, Dose-response

## Abstract

**Background:**

For cesarean delivery (CD), the 90% effective dosage (ED90) of oxytocin for a first bolus has been established. It is not yet known how much oxytocin to inject into obese women undergoing elective discectomy to keep their uterine tone (UT) appropriate. We hypothesized that patients who are overweight need a greater dose of oxytocin infusion; thus, we aimed to determine how the dose-response curve for oxytocin infusion changes following an initial 1 international unit (IU) bolus in obese women undergoing elective CD.

**Methods:**

One hundred parturients with a body mass index (BMI) greater than 30 kg/m^2^ were randomly assigned to receive an infusion rate of 14, 18, 22, or 26 IU/h of oxytocin. When the uterine palpation is as hard as touching the forehead or tip of the nose, it is considered sufficient UT according to the criteria used by obstetricians. The median effective dose (ED50) and ED90 values were determined using probit analysis.

**Results:**

We found the ED50 and ED90 values for the infusion dose of oxytocin were around 11.0 IU/h and 19.1 IU/h, respectively. Each group had a different number of parturients who needed rescued oxytocin: 14 IU/h for six, 18 IU/h for three, one for 22 IU/h, and none for 26 IU/h. The correlation between the frequency of rescued oxytocin administration and the amount of oxytocin infusion needed to avoid uterine atony was statistically significant (*p* = 0.02).

**Conclusion:**

The present research showed that the most effective dosage of oxytocin infusion for obese parturients undergoing elective CD is 19.1 IU/h, following an initial loading dose of 1 IU. Patients with obesity should receive a greater dosage of prophylactic oxytocin, and further studies comparing patients with and without obesity (with higher BMI) are required.

**Clinical Trial Registration:**

https://www.chictr.org.cn/showproj.html?proj=159951, identifier ChiCTR2200059582.

## 1 Introduction

PostPartum hemorrhage (PPH) continues to be the primary cause of maternal mortality throughout the perinatal period. (Rawat et al., 2021). Oxytocin, the primary uterotonic drug, is commonly used to prevent uterine atony. The Royal College of Obstetricians and Gynaecologists in the United Kingdom has advised using oxytocin by delivering it slowly through an intravenous bolus at a dosage of 5 international units (IU). (Prevention and Management of Postpartum Haemorrhage, 2017). Nevertheless, specific clinical studies have demonstrated the efficacy of a lower oxytocin dose in maintaining uterine tone after cesarean delivery. (Carvalho et al., 2004; Sartain et al., 2008; Butwick et al., 2010). To reduce uterotonic drug use and significant obstetric hemorrhage, oxytocin infusion is recommended during cesarean delivery. Thus, a recent global consensus recommends delivering oxytocin in two doses: 1 IU within 15 s, followed by 2.5–7.5 IU per hour during elective cesarean delivery. (Heesen et al., 2019). Oxytocin is linked to significant cardiovascular and noncardiovascular adverse effects, including low blood pressure, rapid heart rate, nausea, and vomiting. (Dyer et al., 2011). Employing the minimal effective dosage to induce sufficient uterine contraction is essential to reduce the negative effects associated with oxytocin.

The incidence of obesity among women in the reproductive age group is on the rise. (Black et al., 2013; Poston et al., 2016). Maternal obesity enhances the likelihood of experiencing pregnancy-induced hypertension, gestational diabetes, cesarean delivery, uterine atony, and increased need for uterotonic medications in comparison to women who are not obese. (Oteng-Ntim et al., 2013; Ma et al., 2016; Butwick et al., 2018). However, oxytocin infusion rates for obese women after elective cesarean delivery to maintain uterine tone remain unclear, even after finding the 90% effective dose (ED90) of oxytocin for an initial bolus. This study examined the dose-response relationship of oxytocin infusion after a 1 IU bolus in obese women during elective CD. The hypothesis was that obese individuals would receive oxytocin infusions higher than the international consensus.

## 2 Materials and methods

### 2.1 Ethics

The randomized, double-blind clinical investigation was conducted at Jiaxing University Affiliated Women and Children Hospital between May 2021 and December 2022 after receiving approval from the Ethics Committee of the hospital (KY-2022-06). We registered this clinical trial at https://www.chictr.org.cn/showproj.html?proj=159951(ChiCTR2200059582). All participants involved in this study provided the written informed consent. The findings of this clinical research were reported by the principles set forth by CONSORT. (Schulz et al., 2010).

### 2.2 Patients and setting

The inclusion criteria are as follows: Individuals classified as American Society of Anesthesiologists physical status class II, aged greater than 18 years, had obesity (BMI ≥30 kg/m^2^), carrying a single fetus at full term (gestation age ≥37 weeks), and women who underwent elective cesarean delivery under spinal anesthesia. This exclusion criteria are as follows: This study did not include women who had preeclampsia or hypertension, other cardiovascular diseases, diabetes or gestational diabetes, malpresentation (such as breech or transverse lie), multiple gestations, previous cesarean delivery, a history of peripartum hemorrhage, placental abnormalities, contraindications to spinal anesthesia, or an allergy to oxytocin. Furthermore, pregnant women in labor who underwent a cesarean delivery were not included in the study.

### 2.3 Study protocol

Following a randomized sheet generated via an online randomization generator (https://www.random.org/sequences/), a research assistant was assigned 100 parturients with a BMI of >30 kg/m^2^ to receive one of four oxytocin infusion rates: 14, 18, 22, or 26 IU/h. This research assistant was in no way involved in other activities. After that, the opaque envelope with the randomized number sheet was opened randomly.

None of the patients were administered premedication. Upon entering the surgery room, a medical professional inserted an 18-gauge intravenous cannula into the left upper arm without administering any prehydration. A noninvasive blood pressure cuff, electrocardiography leads, and pulse oximeter were used to monitor the patient’s vital signs. The L3–L4 vertebral interspace was used for the combined spinal-epidural anesthetic treatment. Patients were positioned left lateral and treated with needle-through-needle. After CSF fluid movement was confirmed, 2.5 mL of hyperbaric 0.5% bupivacaine (10 mg) and 5 μg of sufentanil were injected into the subarachnoid space over 20 s. Lactated Ringer’s solution was injected intravenously at 10 mL/kg/hr of body weight after the intrathecal injection.

Systolic blood pressure (SBP) dropped 20% or greater from baseline, indicating hypotension, with an absolute value of less than 90 mmHg, according to the standards established by our institution. If the systolic blood pressure exceeds 140 mmHg, it is considered hypertension. A heart rate (HR) below 55 bpm is considered bradycardia. If hypotension was present with an HR higher than 90 bpm, 100 µg of phenylephrine was injected intravenously. Conversely, if hypotension happened without an elevated HR, 5 mg of ephedrine was injected intravenously. If atropine was not administered intravenously, phenylephrine infusion would be briefly stopped until the HR was greater than 55 bpm, and therapy could then resume. This was done to address bradycardia and hypotension.

Surgery was authorized once a T5 sensory block to pinprick sensation was verified. A group of highly experienced surgeons specializing in obstetrics consistently carried out cesarean sections, with each physician having more than 15 years of expertise. A gradual intravenous infusion of 1 IU of oxytocin was provided within 15 s after the delivery of the newborn and the clamping of the umbilical cord. To prevent uterine atony, oxytocin was infused continuously at 50 mL/h (adjusted by patient group). A non-participating assistant, knowledgeable about group allocation, prepared oxytocin solutions; the group allocation was concealed from both the researchers (including anesthesiologists and obstetricians) and the patients. Our research protocol involved injecting 3 IU of oxytocin intravenously if the uterine tone (UT) was inadequate 3 min after the initial bolus. In cases where the UT did not improve after two doses of oxytocin, alternative uterotonics were supplied as required, including 0.25 mg intramuscular carboprost or 0.5 mg intravenous sulprostone requested by the obstetrician.

### 2.4 Measurements

The primary outcome of this study was the UT. Assessing the sufficiency of UT for the comprehensive duration of the study was the principal objective of this research. At 3-min intervals, until the peritoneum was closed, the obstetrician, oblivious of the patient groups, assessed the level of satisfaction with the UT. The UT rankings were categorized as highly satisfied (as hard as touching the forehead), moderately satisfied (as hard as touching the tip of the nose), and dissatisfied (as soft as touching the lip). Highly and moderately satisfied UT was regarded as adequate UT. The secondary outcomes included the dose of rescue oxytocin, the requirement for second-line uterine agents, extracorporeal blood volume (EBL), and side effects related to oxytocin. Major postpartum hemorrhage (PPH), defined as extracorporeal blood volume (EBL) exceeding 1000 mL, occurred on or before discharge and was measured by the hemoglobin (Hb) level on the first postoperative day and before discharge; oxytocin-related adverse effects were also considered secondary outcomes. EBL was measured after surgery by a research investigator unaware of the group assignment. To calculate EBL, subtract the initial amniotic fluid volume from the suction bottle’s blood volume, visually estimate the blood in the surgical field and drapes, and subtract the sponges’ dry weight from their weight. (Duffield et al., 2017; Mohta et al., 2021). Blood loss was defined as 1 g for 1 mL regarding weight-to-volume conversion. Acute oxytocin-related reactions include dyspnea, headache, chest discomfort, nausea, vomiting, flushing, and shivering.

### 2.5 Sample size calculation

The Cochran-Armitage test for trend in proportions, part of the PASS^®^ software, was used to estimate the sample size. This test was chosen based on the major outcome measure of the study. Consistent with an initial study with four groups given infusion dosages of 14, 18, 22, and 26 IU/h, the UT improved adequacy rates were determined to be 55%, 75%, 90%, and 98%, respectively. We found a linear trend with a two-sided Z-test at *p* = 0.05, with a 90% power, by enrolling 56 participants in this study. Due to the expected dropouts of 20% of the patient population, each group’s sample size was increased by five patients.

### 2.6 Statistical analysis

We analyzed the distribution of continuous variables using data visualizations and the Kolmogorov−Smirnov test. Statistical analyses were done using SPSS 23.0 (SPSS Inc., Chicago, IL, United States). Data were reported as means ± standard deviation and compared using a one-way analysis of variance, following a normal distribution. Pairwise comparisons were performed using *post hoc* Bonferroni tests. The median (interquartile range) was used and compared for non-normal data using the Kruskal−Wallis test. Pairwise group comparisons were conducted using *post hoc* Dunn’s testing. Categorical variables were compared using Chi-square or Fisher’s exact where appropriate. The Cochran-Armitage χ2 test was performed to evaluate linear trends in parameters among randomly allocated infusion dosage groups, concentrating on the requirement for second-line uterotonics. The oxytocin infusion ED50 and ED95 values were calculated using probit regression. The adequacy of fit between the data and the probit model was assessed using the Pearson chi-square test for goodness-of-fit. Adjusted *p*-values were reported after incorporating Bonferroni corrections. The statistical significance was established using a threshold of two-tailed with *p* < 0.05.

## 3 Results

**FIGURE 1:**
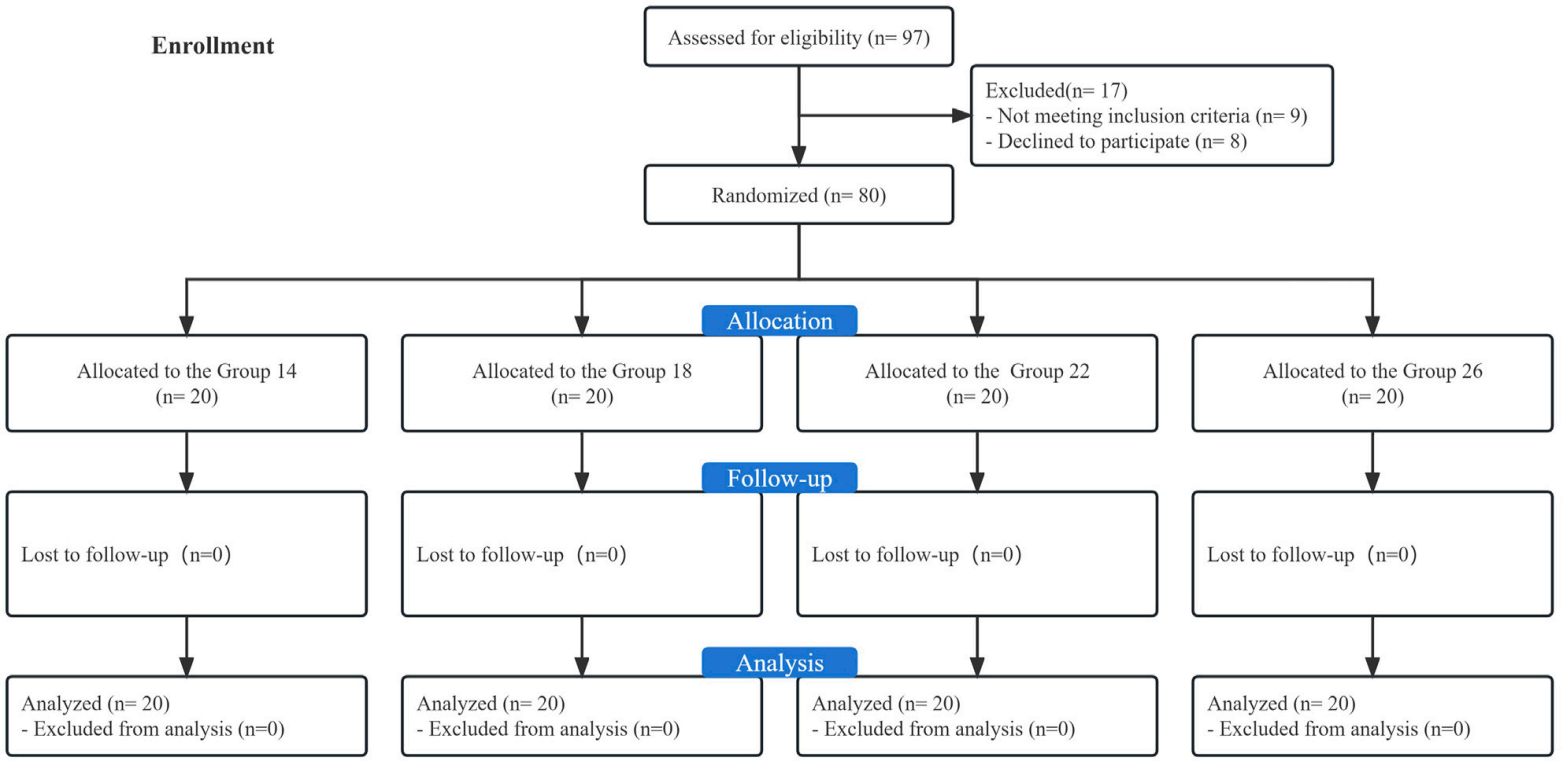
Flow chart of the study.

This study recruited 97 singleton-term women with obesity scheduled for elective CD. Among them, eight declined to participate, and nine were ineligible. The final analysis included the remaining 80 participants (Figure 1). None of the four groups differed significantly from one another in terms of participant characteristics or obstetric data, as presented in Table 1.

**TABLE 1:** Demographics, surgery time, and preoperative Hb.

	Oxytocin infusion rates				
Characteristic	14 IU/h (n = 20)	18 IU/h (n = 20)	22 IU/h (n = 20)	26 IU/h (n = 20)	*p*-Value
Age (year)	30.6 ± 5.0	27.9 ± 3.9	28.7 ± 4.3	28.8 ± 4.7	0.30
BMI (kg/m^2^)	31.9 ± 2.1	33.3 ± 3.9	33.3 ± 2.2	32.2 ± 3.3	0.48
Gestational age (week)	39.4 ± 1.3	38.5 ± 0.8	38.9 ± 1.3	38.7 ± 1.3	0.35
Surgery time (min)	52.3 ± 15.8	54.2 ± 16.8	52.5 ± 14.2	55.7 ± 19.1	0.28
Preoperative Hb (g/dL)	121.30 ± 11.10	120.90 ± 12.42	118.00 ± 12.74	119.18 ± 13.29	0.72
Cesarean history (%)	5 (25)	3 (15)	3 (15)	4 (20)	0.82

Data shown as number (%), or mean (SD) as appropriate.

**FIGURE 2:**
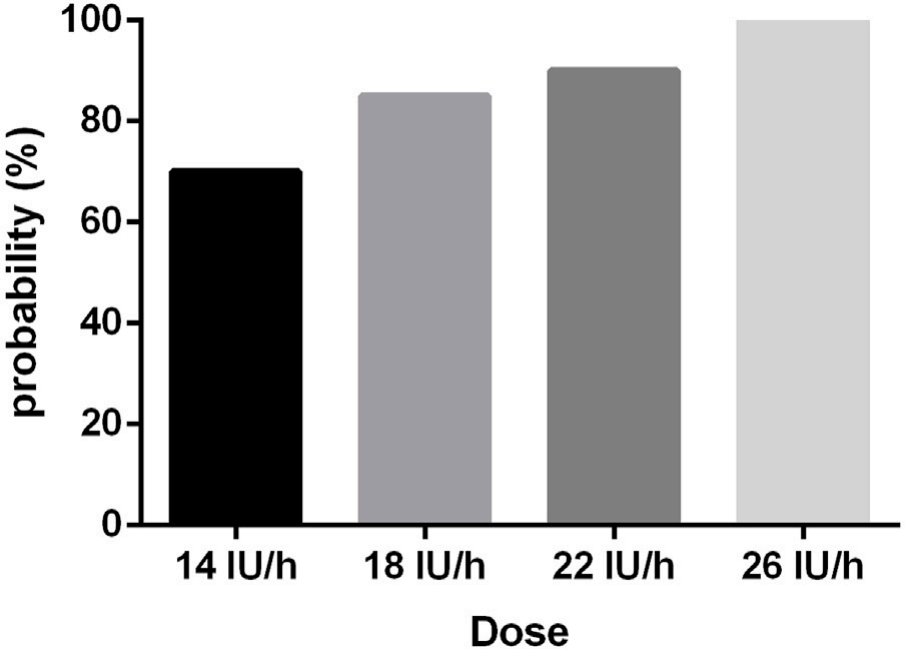
The rates of satisfactory UT in the context of the four different oxytocin infusion doses.

**FIGURE 3:**
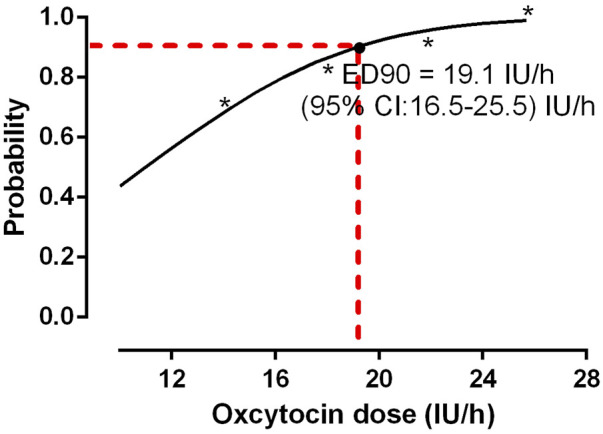
Dose-response curve of oxytocin infusion following a bolus of 1 IU oxytocin. The ED50 and ED90 values for the oxytocin infusion dose needed to avoid uterine atony was around 11.0 IU/h (95% CI: −4.6−4.2) IU/h and 19.1 IU/h (95% CI:16.5–25.5) IU/h, respectively, which was derived from probit analysis.

Figure 2 presents that the rates of satisfactory UT in the context of the four different oxytocin infusion doses, followed by a 1 IU of oxytocin bolus, were 70%, 85%, 95%, and 100%, respectively. Probit analysis was used to determine the estimated ED50 and ED90 values for the oxytocin infusion dose needed to avoid uterine atony, which was around 11.0 IU/h (95% CI: −4.6−4.2) IU/h and 19.1 IU/h (95% CI:16.5–25.5) IU/h, respectively, as depicted in the dose-response curve in Figure 3. The probit model was found to have an excellent match with the data gathered, as evidenced by the goodness-of-fit chi-square test using Pearson’s technique (*p* = 0.864). The groups 14, 18, 22, and 26 had 6, 3, 1, and 0 individuals, respectively, who needed to be rescued with oxytocin during childbirth. A strong correlation was found between the occurrence of parturients needing additional oxytocin and the dosage of oxytocin infusion (*p* = 0.02). The study did not find a statistically significant difference in the occurrence of second-line uterotonics needed among the groups (*p* = 0.10). Table 2 depicts the EBL results for each group, demonstrating no notable distinctions between them (*p* = 0.66). The hemoglobin levels on postoperative day 1 and before discharge showed no statistically significant differences among the groups, as indicated in Table 2. There were no instances of significant postpartum hemorrhage or need for blood transfusion among the individuals included in this study.

**TABLE 2:** Secondary outcomes.

	Oxytocin infusion rates				
	14 IU/h (n = 20)	18 IU/h (n = 20)	22 IU/h (n = 20)	26 IU/h (n = 20)	*p*-Value
Rescue oxytocin required	6 (30)	3 (15)	1 (5)†	0 (0)†	0.02
Secondary uterine agent	3 (15)	1 (5)	0 (0)	0 (0)	0.10
Hb on postoperative day 1 (g/dL)	111.83 ± 9.23	112.93 ± 11.68	113.00 ± 15.45	111.43 ± 15.32	0.66
Hb before discharge (g/dL)	107.63 ± 11.01	111.33 ± 13.42	108.45 ± 13.41	110.36 ± 16.62	0.71
EBL during surgery (mL)	510 (440–765)	570 (465–820)	544 (380–667)	590 (475–720)	0.56

Data shown as number (%), median (quartiles), or mean (SD) as appropriate.

†compared with group 14, *p* values <.05.

Table 3 provides a concise overview of the adverse reactions and outcomes in newborns. These groups did not differ statistically.

**TABLE 3:** Side effects and neonatal Outcomes.

	Oxytocin infusion rates				
	14 IU/h (n = 20)	18 IU/h (n = 20)	22 IU/h (n = 20)	26 IU/h (n = 20)	*p*-Value
Hypotension	5 (25)	4 (20)	5 (25)	6 (30)	0.91
Bradycardia	1 (5)	1 (5)	2 (5)	1 (5)	0.89
Nausea	1 (5)	2 (10)	2 (10)	1 (5)	0.87
Vomiting	0 (0)	1 (5)	0 (0)	1 (5)	0.56
dyspnea	0 (0)	0 (0)	0 (0)	1 (5)	0.39
Flushing	1 (5)	1 (5)	2 (10)	4 (20)	0.34
Headache	0 (0)	0 (0)	1 (5)	0 (0)	0.39
Shivering	0 (0)	1 (5)	0 (0)	1 (5)	0.56
Chest pain	0 (0)	1 (5)	1 (5)	1 (5)	0.39
1-min Apgar score	9 (9–10)	9 (9–10)	10 (9–10)	10 (9–10)	0.54
5-min Apgar score	10 (10–10)	10 (10–10)	10 (10–10)	10 (10–10)	0.90
Umbilical arterial pH	7.33 (7.28–7.36)	7.32 (7.29–7.36)	7.32 (7.28–7.35)	7.33 (7.29–7.37)	0.78

Data shown as number (%), median (quartiles), or mean (SD) as appropriate.

## 4 Discussion

Results from this dose-response trial showed that the effective doses of ED50 and ED90 of preventative oxytocin for keeping UT at a safe level in pregnant women with obesity (defined as a BMI of >30 kg/m^2^) were 11.0 IU/h (95% CI: −4.6−14.2) IU/h and 19.1 IU/h (95% CI:16.5–25.5) IU/h, respectively. Our findings support the idea that obese patients need a higher dose of oxytocin infusion than the 2.5–7.5 IU/h recommended for elective CD in the general population, according to the international consensus. This is consistent with previous research, which found that parturients with a mean BMI of 31.8, comparable to our parturients, had an ED90 of preventive oxytocin of 0.29 IU/min, equals to 17.4 IU/h. (George et al., 2010).

Oxytocin has long been the preferred choice for preventing uterine atony during childbirth. The Royal College of Obstetricians and Gynaecologists in the United Kingdom and the American College of Obstetricians and Gynecologists in the United States of America have jointly established the guidelines for clinical practice. (Carvalho et al., 2004; Sartain et al., 2008; Butwick et al., 2010; George et al., 2010; Schulz et al., 2010; Dyer et al., 2011; Black et al., 2013; Oteng-Ntim et al., 2013; Ma et al., 2016; Poston et al., 2016; Committee on Practice Bulletins-Obstetrics, 2017; Duffield et al., 2017; Prevention and Management of Postpartum Haemorrhage, 2017; Butwick et al., 2018; Heesen et al., 2019; Mohta et al., 2021). Nevertheless, there is currently no universally accepted methodology for the clinical use of oxytocin, particularly in individuals with certain medical problems. Anne Lavoie et al. conducted a study to examine the ED90 for preventing oxytocin infusion in women in labor and women who are not in labor. The results showed that women who have previously received oxytocin from an external source require a greater initial infusion rate of oxytocin than those who have not been exposed to oxytocin before. (Lavoie et al., 2015). Tyagi et al., 2022 evaluated the ED90 of preventive oxytocin infusion in pregnant women with preeclampsia who were also getting magnesium therapy and compared it to normotensive pregnant females. The findings demonstrated a significant rise in the ED90 among persons who were administered magnesium sulfate compared to those who were not (24.9 IU/h vs. 13.9 IU/h).

Obesity is associated with an increased risk of uterine atony and PPH, according to the literature. (Butwick et al., 2018). Consequently, clinical practice stands to gain from any advancements in obesity care. The biased coin up-and-down design was employed by Drew et al., 2020 to examine elective CD procedures in women with a BMI of 40 kg/m^2^ or more. Their findings showed that carbetocin dosages needed by obese women undergoing CD were four times higher than those needed by women without obesity. Peska et al., 2021 tested their initial oxytocin bolus dose in obese and non-obese subjects. A BMI of 40 kg/m2 or above requires more than twice as much oxytocin as a BMI of less than 40 kg/m^2^ (0.78 IU vs. 0.35 IU). However, the ideal oxytocin infusion dosage for obesity is still unknown.

In obese patients (approximately 31 kg/m^2^), the present research analyzed the dose-response curve for prophylactic oxytocin infusion. The findings revealed that the ED50 for elective CD was 11.0 IU/h (the minimal effective dose for reference), whereas the ED90 was 19.1 IU/h (as a comparative dose). This ED90 was significantly higher than the value suggested by a recently published international consensus. As an optimal approach to prevent uterine atony, this consensus suggests administering a starting dose of 1 IU immediately after the birth of the infant, followed by a continuous infusion spanning from 2.5 to 7.5 IU/h. (Heesen et al., 2019). Using an analogous approach, Qian et al. (Qian et al., 2019) demonstrated that the necessary infusion rate for normal-weight parturients to attain an efficacious ED95 was 7.72 IU/h. This value was lower than the outcomes documented in our research, which followed a solitary administration of 1 IU of oxytocin.

We conducted a prospective, double-blind dose-finding study and provided a complete dose-response curve of oxytocin for preventing uterine atony during CD; this is a virtue of the study. A greater degree of individual care information could be derived from the dose-response curve in clinical practice, given that the response of different parturients to oxytocin may vary. The BMI values of the parturients in the present study were marginally greater than the established obesity threshold of 30 kg/m^2^. Thus, the findings of the present study do not explicitly apply to obese parturients with higher BMI values (>35 or 40 kg/m^2^). Further research into the dose-response relationship in this population would be beneficial.

To prevent uterine atony during CD, we determined the initial loading dose to be 1 IU of oxytocin, as supported by the international consensus and the findings of Peska et al., 2021 (Heesen et al., 2019) Both sources recommend 1 IU of oxytocin as an effective loading dose after an infusion. Similar EBL values were observed in all four dose groups of fixed oxytocin infusion. This is because of the careful observation and timely application of supplementary oxytocin and alternative uterine agents as required. Based on the variability in patient responses to oxytocin infusion (which is evident from the results of this study), it is prudent to tailor the management strategy for this medication to the specific needs of each patient.

The frequent assessment of uterine atony at 3-min intervals, along with prompt management, that could interpret that no significant disparities were observed in outcomes related to atony, such as blood loss or changes in hemoglobin levels among the different groups in the current study. It is crucial to note that the administration rate and dosage of oxytocin are crucial factors in determining the severity of cardiovascular adverse effects it can cause, including hypotension, ST depression, and tachycardia. (Heesen et al., 2019). No disparities in the incidence of adverse effects were observed among the cohorts, as determined by our current investigation. It is worth noting that due to the limited sample size in this study and the factors above, statistical power may not have been sufficient to detect variances in secondary outcomes among the groups. This limitation could plausibly explain the observed result.

There are several limitations in this study. First, obstetricians lack a definitive standard to evaluate UT. The assessment of the UT is subjective, rather than an objective quantitative scale. Second, the sample size in this study is inadequate and does not possess the necessary statistical power to effectively evaluate the secondary outcomes. Consequently, these outcomes would be considered as exploratory findings. Third, we did not explicitly analyze the dose-response curve in obese patients compared to those without obesity. Therefore, any comparison with previous research should be approached with caution.

The present results indicated that the optimal oxytocin infusion rate for obese parturients undergoing elective CD is 19.1 IU/h, following a priming dose of 1 IU. It is advisable to administer a greater infusion dose of prophylactic oxytocin to patients who are obese, and additional research is needed to compare this population with those who are obese (with higher BMI values >35 or 40 kg/m^2^).

## Data Availability

The raw data supporting the conclusion of this article will be made available by the authors, without undue reservation.
